# Does psychological support, training and guidance for probation practitioners lead to improved outcomes for service users and staff? A systematic review of Offender Personality Disorder (OPD) Pathway community delivery

**DOI:** 10.1371/journal.pone.0318428

**Published:** 2025-02-13

**Authors:** Aisling O’Meara, Carine Lewis, Jason Davies

**Affiliations:** 1 School of Psychology, Faculty of Medicine, Health and Life Science, Swansea University, Swansea, Wales, United Kingdom; 2 Offender Personality Disorder (OPD) Pathway Programme Team, His Majesty’s Prison and Probation Service, Ministry of Justice, London, United Kingdom; 3 Mental Health and Learning Disability Service Group, Swansea Bay University Health Board, Swansea, Wales, United Kingdom; National Institute of Mental Health and Neurosciences: National Institute of Mental Health and Neuro Sciences, INDIA

## Abstract

This review examines the evidence on outcomes for probation staff and the individuals under their supervision resulting from psychological training and support provided to staff. To be included, papers were required to evaluate impact on the outcomes of workforce development, wellbeing, risk and reconviction, or relationships. This review focussed on the frontline community delivery of a psychologically-informed programme designed for high risk of harm individuals presenting with complex needs; the Offender Personality Disorder (OPD) Pathway. Papers addressing impact of OPD interventions beyond core community delivery, or which addressed unrelated outcomes, were excluded from the review. The databases Scopus and EBSCO were searched on 19th October 2022 and titles were screened for inclusion by two reviewers. Quality of evidence was assessed by the Mixed Methods Appraisal Tool (MMAT). Due to the heterogenous nature of studies included, only narrative knowledge synthesis was possible and this was organised according to area of outcome explored and participant group. Twenty papers were included, comprising ten quantitative, two mixed, and eight qualitative methodologies (including three case studies). Workforce outcomes were the most frequently explored, with impacts noted in relation to self-reported confidence and competence. Impacts regarding risk and reconviction were limited both in relation to the number of studies addressing these outcomes and to the implications that could be drawn from these. Positive relational outcomes were reported as a result of OPD delivery, with consultation and formulation processes leading to better staff-offender relationships. The overall quality of evidence produced in the included studies was of a low to medium standard. Small sample sizes, high attrition rates, bespoke measures, and occasionally questionable analyses were some of the limitations noted. However, taking quality into account, findings were generally indicative of positive impacts of OPD community delivery, although regional differences in delivery model made generalisability of individual findings difficult.

## Introduction

The prevalence of ‘personality disorder’ across the Criminal Justice System is estimated to be between 50 & 70% [1–3]. In 2011, the Offender Personality Disorder (OPD) Pathway was developed; a joint programme between NHS England and the National Offender Management Services (NOMS; now His Majesty’s Prison & Probation Service, HMPPS). This replaced the then Dangerous Severe Personality Disorder (DSPD) programme, aiming to provide a network of services that would reach and potentially help a greater number of individuals [4,5]. The OPD Pathway takes a different approach to rehabilitation, moving away from diagnostic criteria and aims to reduce serious reoffending and improve the psychological health of high risk of harm individuals likely to have a ‘personality disorder’. A key development of this programme is the additional focus on improving workforce competency and wellbeing, in order to help achieve overarching objectives. The OPD Pathway is delivered within both prison and probation settings. Within the community, frontline delivery is known as ‘Core Offender Management’ (Core OM) and outcomes are sought for highly complex people convicted of violent or sexual offences who demonstrate a significant risk of harm and the probation staff managing these individuals.

Differences in the particulars of Core OM delivery across the seven geographical regions have evolved to meet local demands within available local resources, however, several key components underpin all regional interpretations. These include providing probation officers managing individuals who meet OPD Pathway criteria with 1) the tools to identify appropriate individuals for involvement, 2) training in the complexities of ‘personality disorder’ (PD) from a collaboration of psychological and lived experience perspectives (known as the Knowledge and Understanding Framework; KUF), 3) psychological consultation and formulation to inform individual case management, 4) relevant group work (e.g., additional training, group reflective practice sessions), and 5) where deemed necessary, OPD staff jointly working directly with an individual screened into the OPD Pathway and their probation officer [6]. Psychological training and consultation are informed by multiple psychological frameworks including Attachment [7], Schema [8], Desistance [9], and the Good Lives Model [10], with a key focus on building relational practice [11]. The rationale for this review was to draw together the learning across the various regional delivery models in order to attempt to synthesise a clear picture of the evidence of impact that these key components have had on the specified programme outcomes.

While there is a body of literature on various aspects of OPD Pathway service development and delivery, this review examines the evidence for impact of the core community delivery of the OPD Pathway in relation to three of the four stated overarching OPD outcomes [12,13] - workforce development, psychological wellbeing, and reduced risk & reoffending. In addition, the intermediate outcome of relational improvement was also explored, as this has been identified as a key mechanism of change in the programme theory. The fourth OPD outcome, cost effectiveness, has not yet been evaluated in relation to Core OM. The structure and presentation of this review was guided by the Preferred Reporting Items for Systematic reviews and Meta-Analyses (PRISMA) statement S1 Table [14]. This review considers research published from inception of the OPD Pathway in 2012 up to October 2022.

### Objectives

The primary objective of this review was to assess the effects of psychological training and consultation on probation practice and risk management outcomes in relation to both probation staff and the individuals under their supervision. The primary review questions were:

What impact has the OPD Pathway core community delivery had on the:

1. Professional practice approaches and attitudes of probation staff working with individuals screened in to the OPD Pathway?
2. Estimated risk of harm and reoffending or recall rates for those screened into and accessing OPD core community services?
3. Psychological wellbeing and relationships of those screened into and accessing OPD core community services and of the staff managing these individuals?

## Methods

### Protocol registration

The scope of this review was to assess literature that addressed the established outcomes of the OPD Pathway programme [12]. As these outcomes are freely accessible within the OPD Pathway Strategy that was published in 2015, a protocol was not registered for this systematic review. While the absence of a protocol means there is no a-priori public record of the methodological approach, the detail presented in this section provides a transparent account to ensure the ethical integrity of the review in the absence of such a document.

### Eligibility criteria

In order to be eligible for inclusion in the review, papers were required to:

- Address the OPD Pathway core community delivery.
- Report impact (positive, neutral or negative) on the outcomes identified in 1-3 above.
- Be published in English.
- Have a UK affiliation (as the OPD Pathway exists only across England & Wales).

Papers were excluded if they:

- Were based within custodial settings, forensic mental health services (e.g., Medium Secure Units) or if they were resettlement OPD Pathway interventions;
- Did not address impact (e.g., implementation, process) or if the outcomes considered were outside the scope of this review (e.g., satisfaction with service delivery or access to additional services);
- Were not primary research (e.g., reviews or book chapters), anecdotal evidence of impact (i.e., opinion pieces or author view rather than impact for which qualitative or quantitative data were reported).

### Information sources

The electronic databases Scopus (which included full coverage of MEDLINE, EMBASE and Compendex) and EBSCO were searched for the period 01.01.2012 (national introduction of OPD) to 19.10.2022. Additionally, stakeholders in the OPD Pathway (i.e., central leads, regional commissioners, and thematic leads) were contacted for published material that may not have been identified through the search. Four additional titles were suggested by the stakeholders consulted.

### Search strategy

Two separate searches were run to address the two different population groups covered in this review (offenders and probation staff), as detailed in Table 1 with affiliation country limited to UK.

**Table 1:** Search terms utilised for systematic review.

Search	Search terms	N records
Offender outcomes	((TITLE-ABS-KEY (“Offender personality disorder” OR opd) AND TITLE-ABS-KEY (offender* OR criminal* OR probation OR “service user” OR “resident*”)) OR (TITLE-ABS-KEY (formulat* OR “case consultation”) AND TITLE-ABS-KEY (offender* OR criminal* OR probation OR “service user” OR “resident*”) AND TITLE-ABS-KEY (“personality disorder” OR psychopathy)) AND PUBYEAR > 2010) AND (LIMIT-TO (AFFILCOUNTRY, “United Kingdom”)) AND (LIMIT-TO (LANGUAGE, “English”))	95
Workforce outcomes	(TITLE-ABS-KEY (workforce OR staff OR officer OR “offender manager”) AND TITLE-ABS-KEY (offender* OR criminal* OR probation OR “service user” OR “resident*”) AND TITLE-ABS-KEY (competenc* OR development OR attitude OR burnout OR confidence OR understanding OR train* OR supervision OR knowledge OR relat*) AND TITLE-ABS-KEY (“personality disorder” OR opd OR psychopathy) AND PUBYEAR > 2010) AND (LIMIT-TO (AFFILCOUNTRY, “United Kingdom”)) AND (LIMIT-TO (LANGUAGE, “English”))	112

### Selection process

A population-exposure-outcome (PEO) algorithm was employed to guide the selection process:

#### Population.

There were two populations identified (dependent on outcome) for this review: 1) Any individual or group of individuals identified as an offender meeting OPD Pathway criteria and as being on probation; 2) any individual or group of individuals working within probation settings (Offender Managers or Approved Premises staff) and working with individuals identified as meeting OPD Pathway criteria.

#### Exposure.

The offender study population must have been the subject of OPD Pathway consultation or formulation, or have been in receipt of OPD Pathway-probation direct joint working. The staff population must have accessed OPD training, consultations, formulations, group training, or direct joint working with OPD Pathway staff. Study settings must have been in the community (not custody or health settings) and OPD Pathway service delivery must have been within the remit of core OM community delivery.

#### Outcome.

For staff, any outcome related to risk management practices, competence and confidence in professional practice, and wellbeing (including burnout). For offenders, any outcome related to wellbeing, psychological skills development, interpersonal relating, risk of reconviction, risk of harm and community integration. For both groups, improved staff-service user relationships.

All papers were initially screened by two reviewers based on the article title; a review of the abstract was undertaken where it was not readily clear from the title whether a study a) identified impact or b) took place in an OPD Pathway community setting (see Supplemental Materials – DOI 10.17605/OSF.IO/RZ8A5). Reviewers discussed any discrepancies in decisions to reach agreement on initial inclusion. Reviewers then each read the full texts of the first 10 articles that came through the initial title and abstract screening stage. Findings were discussed to reach a satisfactory level of calibration (90%) after which the remaining 201 articles were divided between the reviewers to derive the final set of articles for data extraction.

### Data collection process

To extract and collate data from published literature, an Excel file was designed to capture all relevant fields and to manage referencing. No additional software or systems were employed due to restrictions imposed on adding software to government computers. A detailed extraction chart (see Supplemental Materials - DOI 10.17605/OSF.IO/RZ8A5) was developed to ensure complete and systematic extraction of all relevant data from all included records, with testing of chart fields carried out on the 10 calibration articles. All data were extracted by the first and second authors and a selection of extract records was reviewed by a further reviewer. Where information was lacking in final articles, and where it was unclear whether a study related to the OPD Pathway, article authors were contacted for further information (N = 1). Articles that included subject matter beyond impact (e.g., processes or implementation of services) or unrelated to the specified outcomes had only review-relevant data extracted.

### Data items

The specific outcomes for this review were drawn directly from the overarching OPD Pathway programme policy documentation [12]. These outcomes were:

- Workforce: competence, confidence, psychologically informed practice, improved risk management, improved attitudes towards PD;
- Wellbeing: (specific to service-users) interpersonal skills, thinking skills, coping strategies, prosocial behaviour, happiness, reduced self-harm; (staff-specific) burnout, job satisfaction;
- Risk and reconviction: risk assessments, violent behaviour, recalls, reoffending;
- Relational improvement (in relation to service-users or staff): between staff and service users, peer relations, family relationships.

Changes in recall rates were categorised as a workforce outcome, indicating changes in the use of punitive risk management practices, and as a risk outcome indicating changes in reoffending or offence-paralleling behaviour [15]. Acknowledging that the dynamics of multiple external factors likely affected the interplay between these interpretations of recall rates, findings below were reported in line with their presentation in respective studies.

In addition to outcomes, the contextual variables extracted were: participant demographics (age, gender, offence type {for service users}, time in service {for staff}), participant type (staff or service user), number of participants, group characteristics (where applicable), intervention description, and intervention duration. Research details were also extracted including research design, overall aims, measures used, measurement frequency, analysis used, follow-up duration, alpha values (for psychometrics), themes identified (for qualitative studies), mean difference/change, effect size (for quantitative studies), and direction of effect. Where contextual information was absent or limited, this was acknowledged in the description of findings.

Owing to the highly heterogenous nature of the included studies and the numerous outcomes explored, a formal statistical synthesis of findings was not possible as part of this review. Relatedly, while impact is difficult to definitively evidence through qualitative data, findings relating to relationship outcomes were largely of a qualitative nature. A narrative synthesis approach was thus adopted to summarise the data in relation to each overarching outcome explored.

### Study risk of bias assessment

It was noted that the majority of included studies were undertaken and reported by those commissioned to deliver services. Owing to the nature of the OPD Pathway programme, with few areas having access to dedicated research and evaluation resources, it was necessary to deem this as an acceptable risk of bias, as to exclude studies on this basis would result in little or no material to review. To formally assess risk of bias, the Mixed Methods Appraisal Tool (MMAT [16]) was utilised as this offers a method-specific approach to the critical appraisal of study design and methodology. Through the use of screening questions (S1 - Are there clearly stated research questions?; S2 Do the collected data allow to address the research question?), papers were deemed worthy of critical appraisal if the answer to either or both screening question was “yes”. The four studies failing to meet these criteria were not graded but were retained in the review (see below). The individual appraisals for each paper are presented in the Findings section, with individual method-specific question responses providing insight to decisions made.

### Effect measures

Of the 12 studies utilising a quantitative or mixed methods approach, six employed a range of measures of effect size, reflecting the heterogeneity in research design and analytic approach. In relation to workforce outcomes (e.g., competence, confidence), effects were indicated through the use of Cohen’s d, Cohen’s f, partial Eta squared, and the coefficient of determination (r). In relation to wellbeing, two quantitative studies addressed burnout but only one stated a measure of effect, utilising Cohen’s d. Risk and reconviction rates were measured in five quantitative studies, three of which indicated measures of effect. Cohen’s d, Cohen’s f, and partial Eta squared were provided in these studies. One study utilised a measure of relational improvement once between an intervention and control group, indicating the degree of effect through the coefficient of determination (r). Of the 12 studies incorporating measures of statistical effect in their analyses, the predominant approaches to analysis were tests of group difference (t-tests and ANOVAs). The analyses and effect measures used in each study are presented in tabular form in the Findings section.

### Certainty assessment

In order to evaluate the quality of the evidence contained within each of the included studies, the Mixed Methods Appraisal Tool (MMAT [16]) was applied to each article. For each study type, assessment outcomes were reported as a count of the total of five respective criteria. In relation to screening criterion 1 (Are there clear research questions), articles that reported clear aims or hypotheses in place of research questions were deemed to have met this criterion. The final column in Table 2 indicates the study type (1 – Qualitative; 2 – Quantitative RCT; 3 – Quantitative non-randomised; 4 – Quantitative descriptive; 5 – Mixed Methods) and the total score achieved by those meeting MMAT screening criteria. A score value of NG in this column indicates that the article was not graded due to not meeting initial MMAT screening criteria with further detail of the critical appraisal provided in Table 3. In addition to the four articles that did not meet the MMAT screening criteria required to be graded, seven articles met only two or fewer of the five criteria relevant to their study design. However, all 20 articles were included in this review in order to present as complete a picture as possible of the potential impact of this multifaceted programme of interventions. It should be noted that the data available for analysis in these studies have largely been of a ‘real world’ nature, necessitating convenience sampling of staff and service users operating in dynamic systems where interruptions to organised activity, high turnover and cross-deployment are commonplace. As such, while quality may fall short of the highest scientific standards, the difficulties of conducting research in this field suggest that any insights into the effective functioning of these services may still contribute to the broader evidence base.

**Table 2:** Overview of studies included in systematic review including quality appraisal rating.

Authors	Design/Method	Types of measures	Participants	Analysis and effect measure	Summary findings (in relation to impact on OPDP outcomes)	MMAT*
Blinkhorn, Petalas, Walton, Carlisle, & McGuire (2020)	Qual. Focus groups	Focus groups	Staff = 23 (4 groups of 4-8 participants)	Interpretive Phenomenological Analysis (IPA)	The psychological consultation service was found to build confidence and reassurance in OM’s work and improved their understanding of personality disorder. Improved risk awareness and helpful insights on the importance of the relational process in offender management were also noted by the majority of participants.	Type: 1 Score: NG
Brown, Beeley, Patel, & Völlm (2018)	Mixed. Repeated measures; Open-ended questionnaires	Validated quality checklist; unvalidated questionnaire	Staff = 20 (10% att.)	t-tests	Significant differences were found in 7 of 10 checklist items in relation to case formulation quality after receiving 5 days of training on personality disorder, psychology and case formulation. Significantly higher PDKASQ scores were found post-training on subscales relating to self-assessed competence to work with personality disorder.	Type: 3 Score: 2
Bruce, Horgan, Kerr, Cullen, & Russell (2017)	Quant. Repeated measures and non-equivalent controls	Unvalidated psychometrics; administrative data	Staff = 48; Service users = N not stated	ANOVA	Staff self-reported knowledge and competence in working with PD increased significantly after 1 year of psychologically informed practice. One aspect of burnout significantly improved from baseline to 12-month follow up but no other differences found. A significant decrease in warning and recall rates was reported between pre- and six-months into the intervention while no reduction was seen in the control group.	Type: 3 Score: NG
Bruce, Patel, & Stevens (2020)	Quant. Repeated measures and treatment/ control	Validated and unvalidated psychometrics; administrative data	Staff = 23; Service users = N not stated	ANOVA, Cohen’s f	Staff self-reported knowledge and competence in working with PD increased significantly within 6 months of PIP intervention and in comparison to control data. A measure of burnout showed significant Improvements on one subscale for the intervention group and there was a significant interaction effect between condition and time. No differences were found in offender outcomes post-intervention or in comparison group.	Type: 3 Score: 2
Clark & Chuan (2016)	Quant. Repeated measures	Administrative data	Staff = 10	ANOVA, partial Eta squared	The rate of recalls significantly decreased year on year over three years of intervention for those still receiving input, indicating that psychologically informed practice can reduce officers’ use of recalls for reasons of non-compliance and challenging behaviours while evidencing no increase in serious further offending.	Type: 3 Score: 3
Harvey & Ramsden (2017)	Qual. Single case study	Case study	Staff = 1	Case study	The OM reported being more confident in his probation practice and generalising his learning to other cases. Having increased awareness of interpersonal dynamics led to noticeable changes in his practice by being direct while validating offenders’ vulnerabilities. Improvements in risk management were also reported as a result of psychological consultation and supervision.	Type: 1 Score: NG
Harvey & Sefton (2018)	Qual. Joint case study	Practice observation	Staff = 1; Service user = 1	Case study	After receiving a psychologically-informed warning letter, the offender had fewer police call outs than before, started using the language used within letter to discuss feelings, was more honest and trusting with the OM and the OM reported the relationship had improved.	Type: 1 Score: NG
Jolliffe, Cattell, Raza, & Minoudis (2017a)	Quant. Repeated measures and treatment/ PSM control	Administrative data; risk metrics	Service users = 2092	t-tests, Cohen’s d; Chi^2^	Significant improvements and significant dis-improvements in several criminogenic needs were found for individuals whose OMs had accessed the OPDP as compared to historic data from offender assessments from prior to OPDP implementation. Sub-group comparisons of risk indicated statistically significant improvements for OPDP exposed offenders. This contrasted to clinician-rated risk which increased significantly for OPDP offenders.	Type: 3 Score: 3
Knauer, Walker, & Roberts (2017)	Quant. Non-randomised unmatched samples	Unvalidated questionnaire	Staff = 108	t-tests, Cohen’s d; ANOVA, partial Eta squared	Scores on all questions increased significantly, indicating improvements in self-perceived understanding, confidence, motivation and competence after consultations. Scores did not increase significantly after receiving a formulation letter following the consultation, indicating no additional benefit was perceived as a result of written formulation receipt.	Type: 3 Score: 2
Mapplebeck, Ramsden, Lowton, Short, & Burn (2017)	Qual. Open-ended questionnaire	Unvalidated questionnaire	Staff = 21 (0% att.)	Thematic Analysis	No changes were noted in relation to how probation staff considered risk in relation to case vignettes presented after training. The adoption of a curious stance and a greater focus on offenders’ experiences was noted as positive development in terms of psychological understanding. Slight qualitative differences post-training were noted as potentially additive to existing probation practice.	Type 1 Score: 2
Maltman & Turner (2017)	Qual. Single case study	Case study	Service user = 1	Case study	The application of an OPDP formulation was linked with housing success and plans put in place to reduce anxiety and gain access to external groups. The formulation helped manage emotional responses enabling better relationships with staff.	Type: 1 Score: 4
McMullan, Ramsden, & Lowton (2014)	Qual. Focus groups and interviews	Focus groups and interviews	Staff = 12	Content Analysis	Greater understanding of personality traits and reasons for offending were noted impacts of team consultations on probation practice. A more person-centred and empathetic approach was also identified as a helpful outcome of the process.	Type 1 Score: 3
Minoudis, Craissati, Shaw, McMurran, Freestone, Chuan, & Leonard (2013)	Quant. Repeated measures	Validated quality checklist	Staff = 76 (vignettes -33; practice - 43	t-tests	In relation to one case vignette, formulation training and team consultation did not significantly improve the quality of formulations written by probation staff, however, in relation to the second case vignette, quality checklist scores did improve significantly. In relation to probation case formulations, team consultation over six months had no positive or negative effect on formulation quality scores.	Type: 3 Score: 3
Minoudis, Shaw, & Craissati (2012)	Quant. Exploratory	Administrative data	Service users = 341	Chi^2^; regression analysis	Direct contact with OPD psychologists was negatively associated with community failure and regression modelling indicated an 83% chance of success in the community following direct contact.	Type: 3 Score: 3
Radcliffe, Carrington, & Ward (2020)	Qual. Semi-structured interviews	Semi-structured schedules	Staff = 5	IPA	Improvements in OM’s interpersonal approach to offenders was noted as an outcome of the consultation and formulation process, with greater empathy and increased engagement resulting in better relationships.	Type: 1 Score: 5
Radcliffe, McMullan, & Ramsden (2018)	Quant. Non-randomised matched samples	Validated quality checklist	Staff = 36	Independent t-tests, r coefficient	Significantly higher quality checklist scores were achieved by those in receipt of 6 days formulation training plus ongoing psychological support as compared to OMs who received no training or support. For those trained, longer exposure to OPD support was associated with significantly higher formulation quality, indicating that the ongoing support around real life practice was key to workforce impact.	Type: 3 Score: 2
Ramsden, Lowton, & Joyes (2014)	Quant. Repeated measures	Unvalidated questionnaire	Staff = 46 (74% att.)	Independent t-tests	Significantly higher scores on self-perceived confidence and competence were achieved three months post-consultation intervention for the few who returned responses and statistical analysis was not possible on other measures.	Type: 4 Score: 1
Shaw, Higgins, & Quartey (2017)	Quant. Randomised	Psychometric and rating scale	Staff = 77 (att.-25.6%); Service users = 39	Mann-Whitney U test, r coefficient	On a measure of relationship quality, OMs who collaborated with offenders in case formulation writing had significantly higher scores than controls. Formulation group OMs also scored significantly higher on self-reported confidence in managing their cases. Offenders who collaborated in their formulation writing reported significantly higher trust scores than controls.	Type: 2 Score: 1
Shaw, Minoudis, Craissati, & Bannerman (2012)	Mixed. Repeated measures; Open-ended questionnaires	Validated and unvalidated psychometrics	Staff = 150 (att. not stated but apparent)	t-tests; ANCOVA; thematic analysis	Probation staff’s self-reported understanding of and competency for working with PD was found to significantly improve after one year of a tiered model of consultation, training and supervision, the greatest improvements noted in those receiving the most input. Qualitative responses from 10 staff also indicated improvements in understanding of PD and perceived practice competence.	Type: 5 Score: 3
Webster, Dogget, & Gardner (2020)	Qual. Focus groups and interviews	Semi-structured schedules	Staff = 32	Thematic analysis	Reflective practice group sessions were identified as useful in helping manage job pressures (potentially indicative of preventing burnout) and helped improve psychological approaches to working with complex individuals.	Type: 1 Score: 3

* Type =  1 – Qualitative; 2 – Quantitative RCT; 3 – Quantitative non-randomised; 4 – Quantitative descriptive; 5 – Mixed Methods. Score =  number of MMAT criteria met.

**Table 3:** Risk of bias and critical appraisal of studies as demonstrated through MMAT assessment.

1. Qualitative	S1 Clear RQs	S2 Data allow RQ to be addressed	1.1. Is the qualitative approach appropriate to answer the research question?	1.2. Are the qualitative data collection methods adequate to address the research question?	1.3. Are the findings adequately derived from the data?	1.4. Is the interpretation of results sufficiently substantiated by data?	1.5. Is there coherence between qualitative data sources, collection, analysis and interpretation?
Blinkhorn, et al., 2020	No	Can’t tell	~	~	~	~	~
Harvey & Ramsden, 2017	No	No	~	~	~	~	~
Harvey & Sefton, 2018	No	No	~	~	~	~	~
Maltman & Turner, 2017	Yes	Yes	Yes	Yes	Yes	Yes	No
Mapplebeck et al., 2017	Yes	Yes	Yes	No	Can’t tell	Yes	No
McMullan et al., 2014	Yes	Yes	Yes	Yes	Can’t tell	Yes	Can’t tell
Radcliffe et al., 2020	Yes	Yes	yes	yes	Yes	Yes	Yes
Webster et al., 2020	Yes	Yes	Yes	Yes	Can’t tell	Can’t tell	Yes
**2. Quantitative randomized controlled trials**	**S1 Clear RQs**	**S2 Data allow RQ to be addressed**	**2.1. Is randomization appropriately performed?**	**2.2. Are the groups comparable at baseline?**	**2.3. Are there complete outcome data?**	**2.4. Are outcome assessors blinded to the intervention provided?**	**2.5. Did the participants adhere to the assigned intervention?**
Shaw et al., 2017	Yes	Yes	No	No	No	No	Yes
**3. Quantitative nonrandomized**	**S1 Clear RQs**	**S2 Data allow RQ to be addressed**	**3.1. Are the participants representative of the target population?**	**3.2. Are measurements appropriate regarding both the outcome and intervention (or exposure)?**	**3.3. Are there complete outcome data?**	**3.4. Are the confounders accounted for in the design and analysis?**	**3.5. During the study period, is the intervention administered (or exposure occurred) as intended?**
Brown, et al., 2018	Yes	Yes	Can’t tell	Can’t tell	Yes	Can’t tell	Yes
Bruce, et al., 2020	Yes	Yes	Yes	Yes	No	Can’t tell	Can’t tell
Bruce, et al., 2017	No	No	~	~	~	~	~
Clark & Chuan, 2015	Yes	Yes	Yes	Yes	Yes	Can’t tell	Can’t tell
Jolliffe, et al., 2017	Yes	Yes	Yes	Yes	Yes	Can’t tell	Can’t tell
Knauer, et al., 2017	Yes	Can’t tell	Yes	No	No	No	Yes
Minoudis, et al., 2013	Yes	Yes	Yes	Yes	No	No	Yes
Minoudis, et al., 2012	Yes	Yes	Yes	Yes	Yes	No	can’t tell
Radcliffe, et al., 2018	Yes	Yes	Can’t tell	yes	Yes	No	Can’t tell
**4. Quantitative descriptive**	**S1 Clear RQs**	**S2 Data allow RQ to be addressed**	**4.1. Is the sampling strategy relevant to address the research question?**	**4.2. Is the sample representative of the target population?**	**4.3. Are the measurements appropriate?**	**4.4. Is the risk of nonresponse bias low?**	**4.5. Is the statistical analysis appropriate to answer the research question?**
Ramsden et al., 2014	Yes	No	No	Can’t tell	Yes	No	Can’t tell
**5. Mixed methods**	**S1 Clear RQs**	**S2 Data allow RQ to be addressed**	**5.1. Is there an adequate rationale for using a mixed methods design to address the research question?**	**5.2. Are the different components of the study effectively integrated to answer the research question?**	**5.3. Are the outputs of the integration of qualitative and quantitative components adequately interpreted?**	**5.4. Are divergences and inconsistencies between quantitative and qualitative results adequately addressed?**	**5.5. Do the different components of the study adhere to the quality criteria of each tradition of the methods involved?**
Shaw et al., 2012	Yes	Yes	Yes	Yes	Can’t tell	Yes	No

~Indicates no score due to paper failing to meet screening criteria.

## Findings

### Study selection

**Fig 1:**
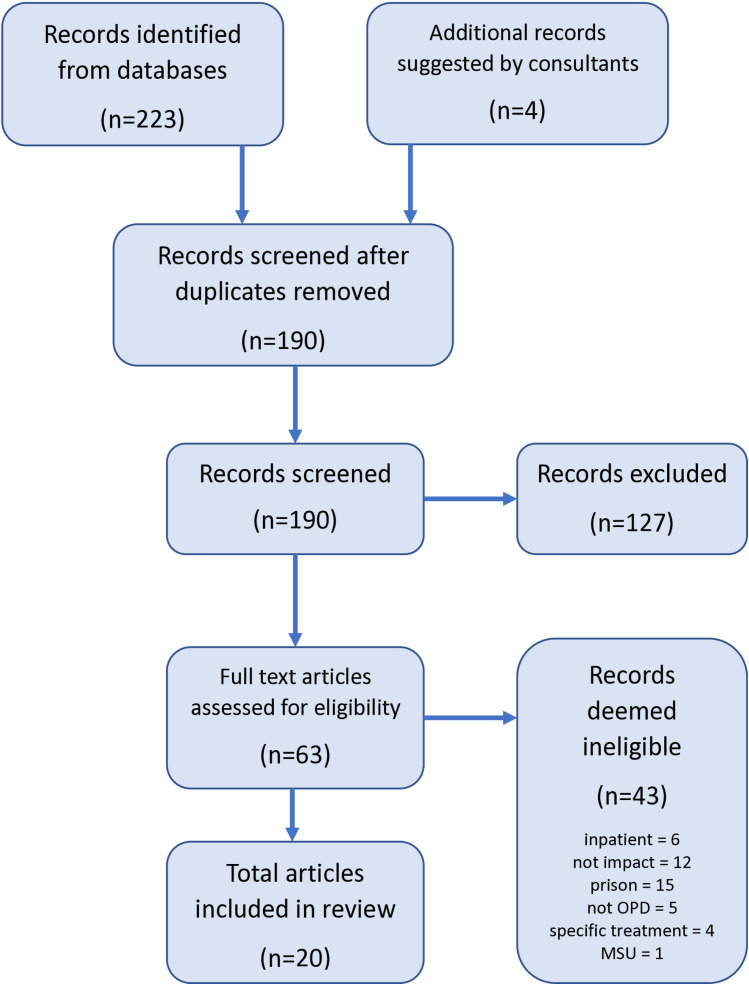
PRISMA flow diagram of article search and selection process.

As shown in Fig 1, there were 207 published articles initially identified in electronic database searches, with key stakeholders of the OPD Pathway programme suggesting a further four articles that did not appear in the search results. Once duplicate entries were removed, all 174 articles were screened according to the criteria set out above, resulting in a final set of 20 papers for inclusion. The majority (n = 19) of these articles were identified in electronic databases with one being identified through the stakeholder route. There were 43 papers whose full-text content precluded inclusion in this review due to inappropriate study population, setting, or service type.

### Study characteristics

Several studies explored the impact of the combination of all components of delivery in tandem, while others attempted to isolate component parts for evaluation. Most frequently isolated components were those of consultation and formulation, however several studies claiming to focus on these also made reference to other components, such as training or group-based supervision. Additionally, as a written formulation is commonly a product of a psychological consultation, many studies treated these concepts as interconnected elements of the service (i.e., a single component). As a result of these anticipated overlaps in component parts of core community delivery, findings from this review were grouped and synthesized according to outcomes rather than to the elements of intervention in order to avoid obvious contamination effects. Consequently, results were grouped first by the outcome explored (workforce (n = 17); wellbeing (n = 4); risk and reconviction (n = 8); and relational improvement (n = 6)), and then by participant group to which they referred (e.g., staff (n = 13), offenders (n = 3), or both (n = 4)). Summary data for each study is presented in Table 2 in alphabetical order by author and MMAT scores are presented in the final column.

### Workforce outcomes

#### Staff participants.

As anticipated, all (n = 13) studies focussing specifically on staff participants and those utilising data from both staff and service user groups (n = 4) explored the impact of OPD Pathway core delivery on key workforce outcomes such as confidence in one’s practice, quality of and ability to write case formulations, approaches to risk management, attitudes towards working with PD and generalisability of learning to the wider caseload.

Competence, Confidence and Attitudes – In quantitative studies, competence, confidence and attitudes were typically explored through the use of the Personality Disorder – Knowledge, Attitudes and Skills Questionnaire (PD-KASQ; [17]), an unvalidated measure of self-reported capabilities and understanding of working with personality disorder. All studies utilising the PD-KASQ [18–22] reported significant increases in competence and confidence for intervention groups (receiving training and consultation) compared to controls or to own baseline, however individual sub-scale differences varied, and three studies did not report effect sizes. Attitudes toward PD did not change in two studies [18,22] while Ramsden et al., reported significantly increased post-intervention attitude scores. Both Bruce et al., studies only reported total scores so specific sub-scale differences cannot be stated [19,20]. Competence was also explored in relation to the quality of case formulations produced by probation staff after receiving targeted training in case formulation writing. The McMurran Formulation Checklist (MFC; [23,24]) was utilised in all three quantitative studies assessing formulation training, and this measure underwent validation assessments as part of the Minoudis et al., [25] study. Training in formulation writing varied in mode and duration of delivery, with some approaches utilising case vignettes alone while others provided continuous practice-based guidance after a dedicated training period. More intensive intervention appeared to be associated with improvement in formulation quality (as rated by the MFC) with Minoudis et al., [25] reporting no change in overall quality after four 2-hour training sessions while Radcliffe et al., [26] reported significant improvements in formulation quality after six days training combined with continuous consultative support.

**Risk management:** Reduced use of recall for reasons other than further offending (e.g., behavioural reasons) were noted in Clark and Chuan [27]. These authors reported a significant reduction in recall rates over time per probation officer, sustained over two years into delivery of a consultation and training model, however no control group findings were reported. Similarly, Bruce et al., [19] reported significantly lower rates of recall and warnings issued by Approved Premises staff who accessed psychologically informed practice (PIP) training and support in comparison to those who did not have access to PIP. However, a slightly modified version of the same intervention found no group differences [20] and poorly matched comparison groups at baseline may account for differences found. Finally, Radcliffe et al., [28] tentatively noted OMs felt consultation services supported their risk management practices, although this was only loosely evident from the qualitative data presented and the authors stated that while risk was always a major focus for OMs, consultations may serve to complement approaches taken.

**Impact on practice:** Qualitative changes in workforce development were also noted as a result of consultation, training, joint working and reflective practice, again with varying degrees of impact. In a case study, consultation and limited joint working were reported to have increased an OM’s self-awareness leading to improved confidence in his practice, an ability to generalise his learning, and better risk management practices [29]. In contrast, six-day personality disorder induction training with 36 probation officers found “only slight qualitative differences” (pp.43) based on two open ended questions [30]. The authors noted that the training resulted in staff taking a more curious stance but that professional priorities, such as understanding one’s history, remained the same as prior to training. Consistent with this, reflective practice sessions for probation staff were seen as useful but as having only an indirect effect on approaches taken to general practice [31], with increased psychological-mindedness reported as the main additive effect. Further, Blinkhorn et al., [32] noted some staff felt increased pressure as a result of involvement with the OPD consultation service and that this service did not contribute to applied operational practices but rather served as an emotionally containing space.

#### Wellbeing.

Despite the OPD Theory of Change [33] making specific reference to improving psychological health, wellbeing, and pro-social behaviour, there were considerable gaps in the literature in relation to direct impact on wellbeing outcomes for either participant group. Wellbeing in relation to staff had not originally been specified in strategic objectives, but has, over the years, evolved to be an intermediary objective in the latest OPD strategy and is a key outcome in the OPD Theory of Change. Staff studies primarily assessed burnout while no offender studies directly addressed features of wellbeing.

#### Staff participants.

Burnout – Although burnout has been identified as one of the major risk factors of working with personality disordered offenders [34], only two quantitative studies and one qualitative study considered this area of staff wellbeing. The Maslach Burnout Inventory (MBI; [35]) was utilised at multiple timepoints in both Psychologically Informed Practice (PIP) studies [19,20]. These showed significant change in the personal accomplishments subscale across the full PIP intervention group and significant post-intervention improvements in emotional exhaustion and depersonalisation for females as compared to male staff. These studies were hampered by shortcomings in design whereby both sets of control groups were maintained only for the first six months. From their qualitative study, Webster et al., [31] tenuously noted that staff who more frequently availed of Reflective Practice (RP) sessions may be protected against the onset of burnout; as a potential knock-on effect of the observed improved ability to manage job pressures reported by those attending RP sessions.

### Risk and reconviction

#### Staff participants.

**Reconviction and recall:** A case study exploring the impact of psychologically informed warning letters on a probationer’s behaviour found considerably fewer police call-outs were made in the months following receipt of the letter compared to the months preceding receipt [36]. Together with findings from the section on risk management above, it is suggested that reducing recall rates may be possible as a result of OPD community delivery, however the most effective approaches to this are yet to be identified.

#### Offender participants.

**Risk:** Both positive and negative findings were reported across different measures of risk for individuals who had some OPD Pathway exposure as compared to a group of historically matched controls (i.e., people who would have screened in to the OPD Pathway had the programme existed at the time of their assessments; [37]). While improvements in four areas of criminogenic need (i.e., dynamic risk factors) were reported, the largest effect size was in relation to a significant, non-desirable change in “lifestyle”. This study also found clinical risk assessments significantly increased in contrast with actuarial risks (based on OASys calculations), which significantly reduced amongst those accessing the OPD Pathway. It is important to note that sample sizes were very small, weakening the power of analyses.

**Reconviction and recall:** Research conducted at the start of the pathway’s introduction showed that a high proportion (48%) of those exposed to OPD involvement were sanctioned for breach of licence conditions or recalled to custody, most commonly for non-compliance [38]. Such rates are perhaps unsurprising given the target group for pathway inclusion. While reoffending and recall rates were reported to differ by geographical area, the authors noted that direct contact with psychologists working within the OPD Pathway was strongly predictive of community success. However, the many different combinations of possible OPD intervention to which participants were exposed made it difficult to identify what aspects may have had direct impacts.

### Relationships

#### Staff and offender participants.

While a core principle of the OPD Pathway is a focus on relationships and the social context in which people live [13], the six studies that examined relationships focussed on the dynamics of the professional context of the OM-offender relationship.

**Relational practice:** All qualitative studies made reference to the role of the OM’s psychological understanding and communication methods in developing relational practice. Three case studies explored the impact on the OM-offender relationships [29,36,39]), all in relation to slightly different compositions of core community delivery. Harvey et al., [29] explored the self-reported impact of the OPD consultation and guidance process, noting that the case study OM became more aware of communication difficulties and through altering his approach, found a greater sense of openness and collaborative working. Maltman & Turner [39] similarly noted how the formulation highlighted to the OM that her own emotional responses could affect probation supervision sessions; adjustments made as a result of this were seen as key to enhancing the OM-offender relationship. This in turn was identified as a protective factor for the offender who had struggled with insecure attachments. Harvey & Sefton’s [36] psychologically informed warning letters intervention was also noted as influential in improving communication between the OM and offender such that a more trusting and honest relationship was built. Two qualitative studies utilising IPA to explore findings from focus groups [32] and interviews [28] in relation to OPD consultations noted the importance of acknowledging difficult dynamics within offender supervision sessions. These studies noted how increased empathy and awareness of an individual’s schemas could lead to improved engagement and more honest interactions [8,10]. Finally, Shaw et al., [40] used a measure of relating with both staff and service users to evaluate the impact of collaborative case formulation in comparison to staff and offenders who did not access formulation processes. Significantly higher overall scores were reported for both formulation groups in comparison to controls, with a measure of working alliance also reaching significance for OMs and scores in relation to trust reported as significantly higher for offenders in the formulation group than those in the control group.

#### Summary findings.

The studies reviewed here suggest that community provision (particularly training, consultation and formulation) can improve feelings of confidence and competence in probation staff. Reflective practice was seen as useful and it was suggested that this may have the potential to be a protective factor for staff wellbeing, however more research is needed to determine this. There was evidence that equipping probation practitioners with better psychological understanding may contribute to improved professional relationships and collaborative working with peers and service users. There was very little evidence however, for the service user outcomes of improved wellbeing and reduced reoffending. While there are indications of improvements in criminogenic needs, this remains a large gap and the complexity of these outcomes will require careful consideration in future research.

#### Certainty of evidence (limitations).

Overall, the quality of evidence presented in the studies included in this review was not of a consistently high standard, as evidenced in the critical appraisal presented in Table 3. Studies failing to meet screening criteria were not scored, as indicated by a ~  in the relevant cells. Scores on the MMAT tended towards low to medium, with only one paper reaching a score of 4/5 and one qualitative paper meeting all five criteria relevant to their study type. For qualitative studies, while approaches used to analyse and gather data were appropriate, several studies failed to meet the criterion of adequately deriving findings from the data. This may have been affected by journal word limits preventing thorough representation of the data, however there were some clearly tenuous interpretations made. The single RCT included in the review failed to meet most criteria, largely due to an absence of reported processes and figures, coupled with a high attrition rate. Of the seven non-randomised quantitative studies assessed, none met the criterion of accounting for confounders in the design and analysis and four failed to demonstrate that the intervention was administered as intended. These studies’ strengths were in relation to appropriateness of data collection and measurement. The two descriptive studies reviewed differed in quality, but both failed to demonstrate a low risk of nonresponse bias and it was not possible to tell if the appropriate analysis methods were used. Finally, one mixed methods study was found to have not met quality standards for each methodological component and the integration of qualitative and quantitative findings were difficult to interpret.

Most quantitative studies appeared to be under-powered (this was not always reported but could be inferred from results) and suffered from high attrition rates. Where significant differences were identified, analyses were often based on small sample sizes or convenience samples (who may not have been representative of the wider population under study, even at a regional level). Additionally, the use of unvalidated psychometrics or bespoke qualitative tools to measure key outcomes warrants caution in interpreting the impact of the intervention under scrutiny, particularly where items were phrased over-simplistically (e.g., “I understand how personality disorder can be linked to offending”). Impact on outcomes reported in many of these studies may be better understood as of a perceived nature rather than as directly evidenced. Additionally, it was possible that post-training responses reflected compliance with training intentions rather than actual learning or development on the part of participants.

The use of historic records to match groups, while necessitated by an absence of contemporary controls, reduced the robustness of the comparisons made. The continuously changing policies governing probation supervision processes and activities suggests historic comparison groups may differ in important ways. Follow-up periods in some studies were quite short or contaminated by exposure to additional elements of service delivery, limiting the scope of impact or strength of comparisons made. Weak design and poorly applied analytical methods meant that methodological rigour was also questionable in several studies. In relation to qualitative findings, all interview and focus group studies relied on self-selected participants, thus the likelihood of positive response biases was high. Those disinclined to engage with the evaluation process may have been those who saw no benefit in the intervention or who did not access interventions offered. Similarly, papers utilising case studies presented somewhat anecdotal evidence, with limited consideration of extraneous factors which may have accounted for the outcomes observed. However, many authors did recognise significant limitations and warned against over-interpretation of findings, instead acknowledging potential directions for further, better designed and more controlled research. While the difficulties of successfully conducting real world research are acknowledged [41], there are a number of useful approaches that practice-based researchers can consider to help mitigate against these pitfalls. For example, embedding well-validated outcome measures into routine practice prior to an intervention can help to ensure measures used are robust and fit for purpose while also enabling a larger and more representative participant sample to be achieved. Maintaining control groups for the duration of an evaluation can ensure useful comparison are made although it is recognised that approaches such as a stepped wedge design may be practically and ethically more appropriate and feasible. Where small-scale designs such as case studies are used, it is important to ensure that rigorous, validated methods are adopted for case study [42] or single case [43] research. Finally, utilising an appropriate research design to fit with the available data and resource can strengthen the overall quality of evaluation undertaken.

In assessing sensitivity of this review to achieved outcomes, a brief sensitivity analysis comparing high- and low-quality studies was conducted. Nine studies achieved medium or high MMAT scores while 11 scored low or could not be graded due to screening criteria. Table 4 presents a comparison of the direction of effect reported in high- vs low-quality studies in relation to each overarching outcome. In relation to workforce outcomes for medium-high scoring studies, some indicated improved competence and a greater understanding of personality, while others noted no or partial effects of training on specific outputs.

**Table 4:** Summary table of brief sensitivity analysis.

Outcome	Medium or high score	NG or low score
Workforce	Mixed positive, negative and no effect findings	Mixed positive, negative and no effect findings
Wellbeing	Only positive findings	Mixed positive and no effect findings
Risk and Reconviction	Mixed positive and negative findings	Mixed positive and no effect findings
Relational Improvement	Only positive findings	Only positive findings

## Discussion

Clearly evidenced within this body of literature was the widely varied approach taken across regional community models to the delivery of psychological consultation and training implemented as part of the OPD Pathway programme. Training programmes were delivered across a variety of different timeframes and to different levels of intensity, from implementing the national KUF programme or building on this with local-level formulation training. Consultations included group discussions, standalone one-to-one meetings, and repeated one-to-one sessions (sometimes jointly with the individual under discussion). Formulations were written exclusively by the psychologist or by an OM after training or in collaboration with the individual on whom the formulation was being written. Generalisability of findings, therefore, may not be applicable beyond the region within which each study was conducted or potentially even beyond the particular setting within which the research was carried out. Standardisation of training materials and resources could ensure a consistently high standard of delivery is achieved. Additionally, the use of file-based practice change data and cross-regional collaboration on delivery could increase the robustness of study designs and consequently the generalisability of the findings produced. Furthermore, it was also not always clear which psychological theories informed the training or consultation delivered, although it is likely that many services opted for hybrid approaches in line with Livesley’s model of integrated treatment [10]. This would be in keeping with policy directives on prioritising relational practice [5] which promote the relevance of attachment theory, the Risk Needs and Responsivity model [44] and desistance theory [9]. However, explicitly documenting the theoretical basis for training and consultation would enable a more detailed understanding of the common and unique theoretical contributions underpinning these activities. Over time, this could help to identify and isolate those theoretical/ model components associated with greater impact. Relatedly, given the multifaceted nature of OPD Pathway community delivery, detailing either 1) which element of delivery achieves specific results or 2) the nature and intensity of each element of intervention received by participants should be considered as part of study design and analysis. Finally, it is relevant to question the flexibility around the specifics of service provision across services and regions. While there are certainly advantages to delivering services adapted to the local resource and demand, this needs to be balanced against the need to deliver a high quality national services. Consequently, the implementation of a standardised core framework of delivery, while allowing scope for add-on modules where relevant, may help build consistency across service delivery and evaluation nationally. This may support a better coordinated focus on achieving specified outcomes across all regions, particularly where consistent recording and reporting practices are introduced.

### Gaps

Across the literature reviewed, notable gaps in the outcomes explored were evident. Workforce outcomes were the most frequently explored, however the quality of studies was low to moderate and, as noted above, unvalidated and potentially biased measures were used to identify changes in competency and in risk management. This would suggest more exploration of this key outcome is warranted. It may be particularly useful to move away from simplistic measures such as increases in PD knowledge and instead explore what features of OPD Pathway community delivery made a useful difference to OM practice. For example, introducing standardised measures of psychologically-informed risk management and relational approaches would be helpful. Work would need to be conducted to compile a selection of validated, fit for purpose measures however, given the issues of measurement identified across the studies in this paper, such an endeavour would be worthwhile. In addition, and given the nuances in OPD delivery (i.e., individually focussed and needs-led based on a case-specific consultation and formulation), more rigorous and replicable qualitative approaches using validated and robust case study, single case and ‘small n’ designs need to be utilised.

In terms of risk management, understanding the mechanisms of change in relation to therapeutic risk taking and what effect this may have on probation supervision processes may help inform service developments. Relatedly, Fitzgibbon et al., [45] noted the importance of integrating clinical judgments with actuarial measures on risk within probation settings in order to ensure an individualised assessment is achieved. One key takeaway from studies demonstrating workforce improvements was that greater exposure to OPD Pathway delivery appeared to achieve greatest impact, thus a particularly useful area of further study would be to more closely examine this potential “dose effect”. It is possible that greater exposure has a greater impact through a broader range of practices being shared and developed over time. For example, a wider array of relational skills use amongst probation staff has been found to lower reconviction rates [46]. It would thus be useful, for example, to understand whether the impact of greater OPD Pathway exposure was related to particularly effective components in relational working espoused by this programme or to individual staff availing of multiple skills-based offerings.

In relation to risk and reconviction, direct impact was not rigorously evaluated in the studies included in this review, although it is appreciated that outcomes of this nature require lengthy study periods in order to allow for appropriate time for relevant events to be observed. Intermediate factors for these outcomes may be more informative, such as reductions in severity of incidents and considerations of mechanisms of change, as touched on by Clark and Chuan [27] in exploring changes to the reasons for recall. Without dedicated objective research resources, longitudinal study design is unlikely in the current OPD Pathway provision, certainly at regional levels. It is also relevant to consider the appropriateness in allocation of individuals to specified intervention programmes prior to exploring related risk and reconviction outcomes [47]. Intermediary outcomes, such as changes in factors known to affect risk levels and reconviction rates, were largely absent in the literature identified for this review, while studies of other OPD services have utilised reasons for recall to custody as an indicative measure of reduced harm, noting reduced seriousness of offending in the reconviction group [48]. A large international body of research on risk and reoffending in relation to offender supervision practices may provide a useful resource for potential approaches to evaluating the impact of psychological interventions offered in the community [46,49]. Such large-scale evaluation of these outcomes is a clear priority for OPD stakeholders, however, barriers such as complex data governance, security and access request processes, difficulty in linking sources of data and a lack of dedicated research staff with the technical resources and know-how to manage available data need to be overcome. Consequently, these barriers to accessing routinely collected organisational-level data at a national or local service level often preclude attempting such studies.

Wellbeing, in spite of being a major intended outcome of the OPD Pathway, was scarcely explored in the reviewed literature. This was particularly evident in relation to offender wellbeing, with just one study addressing coping mechanisms and no other studies addressing any other measure of psychological health or functioning such as skills development, substance use, self-harming or prosocial behaviour. While direct psychometric measurement of these factors is achievable in a prison setting, such self-report measures may be more difficult to gather from a probation cohort, although not impossible [50]. Mixed methods approaches would allow quantitative approaches to be complemented by qualitative investigation of the nuances in wellbeing factors on an individual level. It might be particularly useful to explore mechanisms of change as a result of the psychologically-informed and relational approach promoted by the OPDP (e.g., [11]) in order to determine if, and to what extent, improved wellbeing and mental health results. While studies from within custodial settings such as prison treatment units have provided some insights in this vein [51], the complexities of offender rehabilitation in the community present particular challenges and warrant dedicated attention [52]. The linking of health systems’ data to probation data could significantly help address this gap in our understanding of relevant outcomes in the community. Staff wellbeing was only directly addressed in terms of burnout, while brief mentions of job satisfaction and workplace stress occurred somewhat anecdotally in some qualitative studies. As a result, it was not possible to consider a more granular analysis of which specific components of psychological support, training, consultation or supervision were most effective in this sphere. Clearly this is an area requiring extensive further research especially as within the wider literature on antecedents to correctional staff burnout, few studies address potential interventions to tackle this [53]. Relatedly, the demands on probation staff time and resources necessitate succinct approaches to outcome measurement, thus routinely incorporating general measures of wellbeing and job satisfaction factors may help address the shortfall in literature of this nature (e.g., [54]. In addition research on wellbeing should attend to factors such as staff turnover, sickness, presenteeism and compassion fatigue. Given the continuously changing policies directing probation work, it would be useful to gain an insight into whether and how OPD Pathway processes could help in alleviating the inherent stresses of working in such an unpredictable system.

Finally, relational improvements have been noted as a key goal of the OPD Pathway, and while the OM-offender relationship was explored to some extent, no other element of relationship outcomes were evaluated in the reviewed literature, beyond at an anecdotal level. Burnett et al., discussed in detail the changing nature of probation work over the last few decades and noted the significant shift in attitude toward OM-offender relationships throughout these policy changes [55]. As such, it would be particularly useful to explore the impact and role of OPD services in redeveloping this key process of probation management. To this end, relevant tools (e.g., the Relational Security Explorer or the Working Alliance Inventory) ought to be explored for use in self-evaluation of relational security within OPD services [56,57]. Indeed, in the context of the reunification of probation services, it would be particularly useful to explore the differential impacts on staff wellbeing for those coming from an indirect, phone-based supervision paradigm compared to those with in-person supervision experience.

### Future research

In keeping with the suggestions for further studies already made, future research should attend to the areas of enquiry under evaluation and the approaches to research design and analysis being adopted. The real-world nature of this area of research often presents insurmountable barriers to following a robust scientific design, particularly at a local level. However, through careful construction, a pragmatic use of routine practices, and perhaps a clearer focus on specific inputs (e.g., theories underpinning delivery or a particular service component) or outcomes, it can be possible to generate high quality research content, particularly through utilising realist approaches to evaluation. Some of the difficulties of conducting high quality research in these settings may be overcome by, for example, having an agreed national approach to outcomes monitoring for people on probation. As already noted, this may involve incorporating well-validated measures of key outcomes into routine processes. This would allow the limited resource available for research and evaluation to be targeted at data analysis rather than data collection.

Aligning research design with the underpinning Theory of Change for each service type within the OPD Pathway could also help guide the production of relevant and useful findings that help identify areas of effectiveness and areas for improvement. Clearly documenting inputs, such as theoretical approaches utilised, might help improve the rigour of impact analyses carried out, leaving less room for ambiguity around specifics of service delivery. It is notable, however, that several studies achieved low MMAT scores due to an absence of reporting of steps taken, such as accounting for confounders in design or analysis, or not demonstrating that interventions were administered as intended. Clearer reporting of intervention and analytical processes may have improved scoring considerably in several studies. It may therefore be helpful for those publishing work in this area to use a framework such as MMAT to self-rate their study at the design stage and their paper(s) prior to submission. Relatedly, external collaboration with academic institutions, research councils, or independent researchers, both at a service-specific and national level, may help counteract potential bias in interpretation of findings and increase rigour. Finally, given the considerable differences in the psychological complexities and offending risks presented by male and female OPD service users, future research designs could consider taking a gendered approach to help ensure clarity of outcomes as they relate to each group. As such, it may be useful to explore any differences in how men and women respond to similar OPD interventions, or to evaluate gender-specific services to help inform practice development and potentially identify gender-specific outcomes to explore in future studies. Taken together these steps may help to maximise the quality and robustness of the resulting publications and increase the impact such research can have on service design and practice.

### Limitations of review process

This review was carried out on published literature only and as such, there may be a substantial body of unpublished research which could have contributed to the knowledge base built herein. It is also acknowledged that search terms employed did not clearly map onto all outcomes explored, particularly in relation to offender search terms where specific outcomes of reoffending, risk, wellbeing, etc., were not utilised. Search terms were developed to ensure the correct participant groups were captured and less priority was given to specified outcomes to avoid potentially missing content that used vague or indicative language. Additionally, while the initial screening and selection stages of this review were conducted by two researchers, the majority of the extraction and all of the synthesis process was carried out by the first author alone. As a result, the conclusions drawn and quality assessments made were not subject to any formal external validation processes. Future reviews of this nature would benefit from wider collaboration with independent reviewers to strengthen reliability and avoid potential bias in interpretation of findings. It is further acknowledged that a protocol for this review was not registered in advance of carrying out the study. While protocol registration is increasingly used to provide an assurance that reviewers will not stray from their intended scope, assurance regarding scope was provided by the previously published and publicly available OPD Strategy [12] which describes the nature of Core OM delivery and the intended outcomes of these services. Further, the detailed description of the method presented above is intended to allow replication of the study conducted and to provide assurance as to the methodological integrity of this analysis. It is relevant to note that higher quality study design in the reviewed papers may have allowed for the identification of common themes and contrasting insights derived from different methodological approaches, however such comparison of findings by research design was not possible in this study.

### Implications for practice and policy

The reviewed literature presented too little in terms of concrete outcomes directly resulting from OPD Pathway interventions to be informative about clear directions for criminal justice policy and probation practice, however, there were some useful indicative findings. Overall, there was some support for the provision of psychological guidance and consultation processes being helpful to probation staff in terms of their direct working with complex individuals. Gaining insights into the historic factors that influence behavioural responses to perceived triggers and learning to utilise more psychologically informed communication methods with their service users may help probation staff to identify and mitigate against potential risk factors for further offending or harmful behaviours. Two qualitative staff studies made brief reference to the additional pressure felt by OMs as a result of OPDP involvement. This raises the pertinent question of potential unintended or iatrogenic effects on staff (such as compassion fatigue) experienced as a result of developing a psychologically informed approach and understanding of offending behaviour. It may therefore be prudent to explore in more detail the implications of adopting relational ways of working, and the nature of the training, and supervision required for staff to enable and sustain this way of working. Indeed, staff supervision and reflective practice are increasingly being recognised as an essential component for the safe and effective delivery of services [58]. Perhaps the integration of frameworks such as bio-psycho-social theory and trauma-informed practice into the established probation qualification training programme may also support this development.

## Conclusion

This systematic review sought to assess the effectiveness of OPD training and consultation on probation practice, risk and reoffending, wellbeing and OM-offender relationships. The quality of evidence reviewed was of a low to medium standard overall, making it difficult to arrive at clear answers to review questions. Small sample sizes, high attrition rates, bespoke measures, and occasionally questionable analyses were some of the limitations noted. However, taking quality into account, the trends within the findings were generally indicative of positive impacts of OPD community delivery, although regional differences in delivery model made generalisability of individual findings difficult. Several directions for future research and policy implications have been suggested.

## Supporting information

S1 Table PRISMA 2009 checklist. (DOC)
