# Supplementary material for: Does psychological support, training and guidance for probation practitioners lead to improved outcomes for service users and staff? A systematic review of Offender Personality Disorder (OPD) Pathway community delivery
Source: PLoS One. 2025 Feb 13;20(2):e0318428. doi: 10.1371/journal.pone.0318428 (PMC11824987; doi:10.1371/journal.pone.0318428)
Supplement: Attachment — Submitted filename: Response to Reviewers.docx [file pone.0318428.s002.docx]

Response to Reviewers

Dear Reviewers,

Thank you very much for taking the time to review our manuscript. We greatly appreciated your feedback and have done our best to make amendments in line with your suggestions. We were particularly thankful for your acknowledgement of the importance of this work and hope that you see fit to recommend the revised paper for publication.

In particular, the following changes have been made in tracked changes:

**Reviewer #1:** The topic seems to be a really good one.
1) In the discussion section, it should be explored a bit more with contrasts and comparisons including some citations.

Additional discussion points have been included to address wider literature on the outcomes explored.

2) The grammatical and syntax errors need to be corrected

Typos have been addressed.

**Reviewer #2:** This is a systematic review on a noble and important topic. However, in my opinion, there are lots of major issues associated with this study.
1. The authors have not mentioned about the registration of the PROTOCOL of the systematic review; this is required nowadays before conducting this type of study. Example: registration in PROSPERO. This is a major ethical aspect lacking in this study

We have included a paragraph in the Methods section to explain the absence of a protocol and to direct readers to a published strategy document which guided the parameters of this review. We have also added a paragraph to the limitations section of the Discussion to address the ethical concerns around not providing a protocol.

2. The authors have not described about the tools used in screening process. Eg. Covidence, Citation manager, etc.

Section 2.6 ‘Data collection process’ has been updated to include reference to the tools utilised in this review.

3. The manuscript should contain a table depicting qualitative analysis of the included studies. This is lacking here

Table 2 provides a narrative account of the included studies and broadly covers the authors’ quality assessment. Table 3 has been added to provide a more detailed account of the authors’ critical appraisal of the studies included in this review.

4. The Risk of bias (RoB) assessment need to be done even though the studies carry high risk of bias. This is an essential component of systematic review.

The risk of bias was assessed through the use of the Mixed Method Appraisal tool and an additional table has been inserted to provide greater detail on the considerations made in relation to each paper. This critical appraisal tool has been noted as an established RoB tool.

5. It would be better if the quantitative findings are presented in a table.

Detailed findings have been documented in the study data, which will be made available to readers alongside publication.

We hope you find these amendments and responses satisfactory and that you will consider this manuscript acceptable for publication in light of these changes.

Thank you, again, for your time and consideration.

Kind regards,

Aisling O’Meara, Carine Lewis, & Jason Davies
