# Supplementary material for: Does psychological support, training and guidance for probation practitioners lead to improved outcomes for service users and staff? A systematic review of Offender Personality Disorder (OPD) Pathway community delivery
Source: PLoS One. 2025 Feb 13;20(2):e0318428. doi: 10.1371/journal.pone.0318428 (PMC11824987; doi:10.1371/journal.pone.0318428)
Supplement: Attachment — Submitted filename: PLOS REVIEW DOCUMENT.docx [file pone.0318428.s003.docx]

**Review Comments: -**

The authors have conducted a thorough review with clear identification of gaps in the literature and directions for future research. However, several areas could be improved to enhance the clarity and impact of the manuscript:

1. **Clarification of Psychological Theories and Models**: The authors mention psychological consultation and training, but it is unclear which psychological theories informed the training. It would be beneficial to emphasize the importance of consistently identifying these theoretical foundations across studies. Discussing how different theories impact outcomes could guide future research and service development.
2. **Generalisability and Standardization**: The discussion about generalisability could be expanded. The authors should suggest how future studies could improve on this limitation, possibly by proposing a standardized framework for the OPD Pathway. This would ensure more consistent delivery across regions and allow for better comparison and evaluation of outcomes.
3. **Addressing Methodological Gaps**: The manuscript acknowledges that some studies failed to meet quality standards. The authors should propose specific methodological improvements, such as encouraging the use of validated measures, increasing rigor in evaluation designs, or integrating longitudinal methods. Providing specific examples or frameworks for improving study design would enhance future research.
4. **Focus on Offender Wellbeing**: Offender well-being was scarcely explored in the studies reviewed. The authors could propose more detailed methodologies for incorporating well-being measures into future studies, particularly through qualitative approaches. This would make the review more actionable for researchers expanding this area.
5. **Practical Recommendations for Policymakers**: The "Implications for Practice and Policy" section could be strengthened by offering specific suggestions for policymakers. This could include recommending key areas where psychological training should be mandated and tools such as the Relational Security Explorer for evaluating relational security.
6. **Collaborative Validation**: The authors could recommend that future reviews use a more collaborative validation process involving multiple independent reviewers. This would reduce potential bias and increase the reliability of findings.
7. **Expanding on Gendered Approach**: The review suggests exploring gendered differences in psychological complexities. The authors could offer more detailed examples of how a gendered approach could be applied, such as examining how male and female service users respond differently to the OPD Pathway and proposing specific outcome measures that may vary by gender.
8. **Risk and Reconviction Data**: The manuscript highlights limited data on risk and reconviction outcomes, which are critical indicators of program success. The authors could explore why this gap exists and suggest ways to encourage future research to focus on these outcomes. A more detailed analysis or explanation for the limited data would improve the discussion.
9. **Workforce Outcomes**: The review finds improvements in workforce outcomes, such as confidence and competence, but could benefit from a more granular analysis. The authors could explore which specific components of psychological support and training were most effective, offering insights into what works best.
10. **Clarify the Impact of Low-Quality Studies**: Including lower-quality studies is acknowledged but should be clarified. A sensitivity analysis comparing higher- and lower-quality studies could provide more nuanced conclusions about the robustness of the evidence.
11. **Enhancing the Conclusion with Practical Implications**: The conclusion could be strengthened by discussing concrete implications for practitioners and policymakers. The authors could highlight what probation services should implement based on these findings and how future interventions should be designed to maximize their impact.
12. **Improving the Synthesis of Quantitative and Qualitative Data**: Since the review includes both quantitative and qualitative studies, the authors should provide a more integrated synthesis of the findings from these two approaches. Highlighting common themes or contrasting insights would give readers a fuller understanding of how different types of evidence contribute to the overall conclusions.
