# Supplementary material for: Does psychological support, training and guidance for probation practitioners lead to improved outcomes for service users and staff? A systematic review of Offender Personality Disorder (OPD) Pathway community delivery
Source: PLoS One. 2025 Feb 13;20(2):e0318428. doi: 10.1371/journal.pone.0318428 (PMC11824987; doi:10.1371/journal.pone.0318428)
Supplement: Attachment — Submitted filename: Response to reviewers 2024.docx [file pone.0318428.s004.docx]

**Response to reviewers**

Sincere thanks to both reviewers for their helpful and constructive feedback. We have endeavoured to address all comments by taking the following specific actions:

Reviewer 3

As suggested, a separate conclusion aligned to objectives of the review has been added.

While a protocol was not prepared, the lines of enquiry for this systematic review were informed directly by the OPD strategy which was published in 2015. This document has been referenced in the manuscript and a paragraph in the Methods section informs readers of where to access this strategy. The concerns around absence of a protocol are also addressed in the ‘Discussion’ section.

We have added a line to the ‘Study selection’ section to indicate the number of studies appearing to meet the criteria but excluded from the review.

Reviewer 4

1.	Clarification of Psychological Theories and Models:

Specific reference to psychological models informing OPD service delivery have been made throughout the manuscript. We have also addressed the integration of models in service delivery and noted the difficulty in determining direct impact of individual models on outcomes as a result of this integration. We have, however, provided some suggestions in the ‘Future research’ section for clearer identification of theoretical approaches utilised.

2.	Generalisability and Standardization:

We have expanded the discussion on generalisability and made direct suggestions for improvements to service delivery and data collection practices that will help make findings from future studies more generalisable. The reviewer’s suggestion to propose a standardised framework for the OPD Pathway was followed.

3.	Addressing Methodological Gaps:

All suggestions on discussing methodological gaps were addressed and incorporated throughout the ‘Findings’ and ‘Discussion’ sections.

4.	Focus on Offender Wellbeing:

More attention has been given to the discussion of wellbeing outcomes and specific suggestions for accessing more relevant wellbeing data were made.

5.	Practical Recommendations for Policymakers:

The reviewer’s specific suggestions for mandating training and tools use were followed and more direct implications for policy and practice were added.

6.	Collaborative Validation:

We have added the suggestion to involve independent reviewers in future reviews of this nature to the ‘Limitations in the review process’ section and have noted this would improve reliability of findings.

7.	Expanding on Gendered Approach:

We have expanded on the discussion of taking a gendered approach in future studies and incorporated the reviewer’s specific suggestion to propose examining how male and female service users respond differently to the OPD Pathway.

8.	Risk and Reconviction Data:

We have provided some further insights into the absence of good quality risk and reconviction data and discussed the complexities around access to this kind of information, both on a service level and a national level. Specific points around difficulties in data linking and access requirements were included to help elaborate on this issue.

9.	Workforce Outcomes:

Granular analysis of workforce outcomes was not possible in this review as it was not generally clear in the published studies what aspects of training or consultation were effective. We have given some space to discussion around the interwoven nature of service delivery and the benefits of having a clearer framework against which to monitor outcomes. Linked with points 1 and 2, we have made suggestions for standardisation of services and training and better targeted outcomes monitoring. It is proposed that implementing more structured frameworks might help future studies address what works best in these services.

10.	Clarify the Impact of Low-Quality Studies:

A section on sensitivity has been included in the ‘Certainty of evidence (Limitations)’ section and a brief summary table comparing the direction of effect found in high and low quality studies has been added.

11.	Enhancing the Conclusion with Practical Implications:

Specific discussion points around practical implications were added and these provided suggestions for consistent service delivery which should help identify what works in this field of work. Proposals for service improvements in line with these suggestions have been made.

12.	Improving the Synthesis of Quantitative and Qualitative Data:

We have noted in the ‘Limitations in the review process’ section that higher quality study design may have allowed for a better synthesis of findings across qualitative and quantitative papers but that a sophisticated synthesis of themes was not possible with the studies reviewed.
