# Supplementary material for: Does psychological support, training and guidance for probation practitioners lead to improved outcomes for service users and staff? A systematic review of Offender Personality Disorder (OPD) Pathway community delivery
Source: PLoS One. 2025 Feb 13;20(2):e0318428. doi: 10.1371/journal.pone.0318428 (PMC11824987; doi:10.1371/journal.pone.0318428)
Supplement: Attachment — Submitted filename: REVIEWER COMMENTS plos.docx [file pone.0318428.s005.docx]

REVIEWER COMMENTS!

Accept the manuscript, as the authors have addressed all queries.
