# Supplementary material for: Does psychological support, training and guidance for probation practitioners lead to improved outcomes for service users and staff? A systematic review of Offender Personality Disorder (OPD) Pathway community delivery
Source: PLoS One. 2025 Feb 13;20(2):e0318428. doi: 10.1371/journal.pone.0318428 (PMC11824987; doi:10.1371/journal.pone.0318428)
Supplement: Peer review history

# Author comment: Author response to Decision Letter 0

**DOI:** 10.1371/journal.pone.0318428.r001

4 Aug 2023

# Peer review: Decision Letter 0

**DOI:** 10.1371/journal.pone.0318428.r002

**Authors:** Nimesh Lageju

27 Sep 2023

PONE-D-23-24839

Does psychological support, training and guidance for probation practitioners lead to improved outcomes for service users and staff? A systematic review of Offender Personality Disorder (OPD) Pathway community delivery

PLOS ONE

Dear Dr. O'Meara,

Thank you for submitting this article and trusting this journal for publication. I really appreciate the efforts of all the authors in preparing this manuscript. The authors have conducted a systematic review on crucial and significant topic. However, I regret to inform the authors that I am unable to accept this manuscript at its current condition. The authors have not mentioned the systematic review protocol's registration, which is currently required for carrying out such studies, such as signing up in PROSPERO. This absence raises significant ethical concerns concerning this study.

I am sorry that we cannot be more positive on this occasion, but hope that you appreciate the reasons for this decision.

Kind regards,

Nimesh Lageju

Academic Editor

PLOS ONE

Reviewers' comments:

Reviewer's Responses to Questions

**Comments to the Author**

1. Is the manuscript technically sound, and do the data support the conclusions?

The manuscript must describe a technically sound piece of scientific research with data that supports the conclusions. Experiments must have been conducted rigorously, with appropriate controls, replication, and sample sizes. The conclusions must be drawn appropriately based on the data presented.

Reviewer #1: Yes

Reviewer #2: Partly

**********

2. Has the statistical analysis been performed appropriately and rigorously?

Reviewer #1: N/A

Reviewer #2: N/A

**********

3. Have the authors made all data underlying the findings in their manuscript fully available?

The PLOS Data policy requires authors to make all data underlying the findings described in their manuscript fully available without restriction, with rare exception (please refer to the Data Availability Statement in the manuscript PDF file). The data should be provided as part of the manuscript or its supporting information, or deposited to a public repository. For example, in addition to summary statistics, the data points behind means, medians and variance measures should be available. If there are restrictions on publicly sharing data—e.g. participant privacy or use of data from a third party—those must be specified.

Reviewer #1: Yes

Reviewer #2: Yes

**********

4. Is the manuscript presented in an intelligible fashion and written in standard English?

PLOS ONE does not copyedit accepted manuscripts, so the language in submitted articles must be clear, correct, and unambiguous. Any typographical or grammatical errors should be corrected at revision, so please note any specific errors here.

Reviewer #1: Yes

Reviewer #2: Yes

**********

5. Review Comments to the Author

Please use the space provided to explain your answers to the questions above. You may also include additional comments for the author, including concerns about dual publication, research ethics, or publication ethics. (Please upload your review as an attachment if it exceeds 20,000 characters)

Reviewer #1: The topic seems to be a really good one.

1) In the discussion section, it should be explored a bit more with contrasts and comparisons including some citations.

2) The grammatical and syntax errors need to be corrected

Reviewer #2: This is a systematic review on a noble and important topic. However, in my opinion, there are lots of major issues associated with this study.

1. The authors have not mentioned about the registration of the PROTOCOL of the systematic review; this is required nowadays before conducting this type of study. Example: registration in PROSPERO. This is a major ethical aspect lacking in this study

2. The authors have not described about the tools used in screening process. Eg. Covidence, Citation manager, etc.

3. The manuscript should contain a table depicting qualitative analysis of the included studies. This is lacking here

4. The Risk of bias (RoB) assessment need to be done even though the studies carry high risk of bias. This is an essential component of systematic review.

5. It would be better if the quantitative findings are presented in a table.

**********

6. PLOS authors have the option to publish the peer review history of their article (what does this mean? ). If published, this will include your full peer review and any attached files.

If you choose “no”, your identity will remain anonymous but your review may still be made public.

**Do you want your identity to be public for this peer review?** For information about this choice, including consent withdrawal, please see our Privacy Policy .

Reviewer #1: No

Reviewer #2: No

**********

[NOTE: If reviewer comments were submitted as an attachment file, they will be attached to this email and accessible via the submission site. Please log into your account, locate the manuscript record, and check for the action link "View Attachments". If this link does not appear, there are no attachment files.]

- - - - -

For journal use only: PONEDEC3

# Author comment: Author response to Decision Letter 1

**DOI:** 10.1371/journal.pone.0318428.r003

4 Dec 2023

Dear Reviewers,

Thank you very much for taking the time to review our manuscript. We greatly appreciated your feedback and have done our best to make amendments in line with your suggestions. We were particularly thankful for your acknowledgement of the importance of this work and hope that you see fit to recommend the revised paper for publication.

In particular, the following changes have been made in tracked changes:

Reviewer #1: The topic seems to be a really good one.

1) In the discussion section, it should be explored a bit more with contrasts and comparisons including some citations.

Additional discussion points have been included to address wider literature on the outcomes explored.

2) The grammatical and syntax errors need to be corrected

Typos have been addressed.

Reviewer #2: This is a systematic review on a noble and important topic. However, in my opinion, there are lots of major issues associated with this study.

1. The authors have not mentioned about the registration of the PROTOCOL of the systematic review; this is required nowadays before conducting this type of study. Example: registration in PROSPERO. This is a major ethical aspect lacking in this study

We have included a paragraph in the Methods section to explain the absence of a protocol and to direct readers to a published strategy document which guided the parameters of this review. We have also added a paragraph to the limitations section of the Discussion to address the ethical concerns around not providing a protocol.

2. The authors have not described about the tools used in screening process. Eg. Covidence, Citation manager, etc.

Section 2.6 ‘Data collection process’ has been updated to include reference to the tools utilised in this review.

3. The manuscript should contain a table depicting qualitative analysis of the included studies. This is lacking here

Table 2 provides a narrative account of the included studies and broadly covers the authors’ quality assessment. Table 3 has been added to provide a more detailed account of the authors’ critical appraisal of the studies included in this review.

4. The Risk of bias (RoB) assessment need to be done even though the studies carry high risk of bias. This is an essential component of systematic review.

The risk of bias was assessed through the use of the Mixed Method Appraisal tool and an additional table has been inserted to provide greater detail on the considerations made in relation to each paper. This critical appraisal tool has been noted as an established RoB tool.

5. It would be better if the quantitative findings are presented in a table.

Detailed findings have been documented in the study data, which will be made available to readers alongside publication.

We hope you find these amendments and responses satisfactory and that you will consider this manuscript acceptable for publication in light of these changes.

Thank you, again, for your time and consideration.

Kind regards,

Aisling O’Meara, Carine Lewis, & Jason Davies

Attachment Submitted filename: Response to Reviewers.docx

# Peer review: Decision Letter 1

**DOI:** 10.1371/journal.pone.0318428.r004

**Authors:** Pracheth Raghuveer

11 Oct 2024

PONE-D-23-24839R1Does psychological support, training and guidance for probation practitioners lead to improved outcomes for service users and staff? A systematic review of Offender Personality Disorder (OPD) Pathway community deliveryPLOS ONE

Dear Dr. O'Meara,Thank you for submitting your manuscript to PLOS ONE. After careful consideration, we feel that it has merit but does not fully meet PLOS ONEs publication criteria as it currently stands. Therefore, we invite you to submit a revised version of the manuscript that addresses the points raised during the review.Please submit your revised manuscript by 10th October 2024. If you will need more time than this to complete your revisions, please reply to this message or contact the journal office at plosone@plos.org . When you're ready to submit your revision, log on to https://www.editorialmanager.com/pone/ and select the 'Submissions Needing Revision' folder to locate your manuscript file.

Please include the following items when submitting your revised manuscript:

- A rebuttal letter that responds to each point raised by the academic editor and reviewer(s). You should upload this letter as a separate file labeled 'Response to Reviewers'.
- A marked-up copy of your manuscript that highlights changes made to the original version. You should upload this as a separate file labeled 'Revised Manuscript with Track Changes'.
- An unmarked version of your revised paper without tracked changes. You should upload this as a separate file labeled 'Manuscript'.

If you would like to make changes to your financial disclosure, please include your updated statement in your cover letter. Guidelines for resubmitting your figure files are available below the reviewer comments at the end of this letter.

If applicable, we recommend that you deposit your laboratory protocols in protocols.io to enhance the reproducibility of your results. Protocols.io assigns your protocol its own identifier (DOI) so that it can be cited independently in the future. For instructions see: https://journals.plos.org/plosone/s/submission-guidelines#loc-laboratory-protocols . Additionally, PLOS ONE offers an option for publishing peer-reviewed Lab Protocol articles, which describe protocols hosted on protocols.io. Read more information on sharing protocols at https://plos.org/protocols?utm_medium=editorial-email&utm_source=authorletters&utm_campaign=protocols .

We look forward to receiving your revised manuscript.

Kind regards,

Pracheth Raghuveer, MD, DNB

Academic Editor

PLOS ONE

Journal Requirements:

When submitting your revision, we need you to address these additional requirements.1. Please ensure that your manuscript meets PLOS ONE's style requirements, including those for file naming. The PLOS ONE style templates can be found at https://journals.plos.org/plosone/s/file?id=wjVg/PLOSOne_formatting_sample_main_body.pdf and https://journals.plos.org/plosone/s/file?id=ba62/PLOSOne_formatting_sample_title_authors_affiliations.pdf2. We note that you have stated that you will provide repository information for your data at acceptance. Should your manuscript be accepted for publication, we will hold it until you provide the relevant accession numbers or DOIs necessary to access your data. If you wish to make changes to your Data Availability statement, please describe these changes in your cover letter and we will update your Data Availability statement to reflect the information you provide.3. Please note that in order to use the direct billing option the corresponding author must be affiliated with the chosen institute. Please either amend your manuscript to change the affiliation or corresponding author, or email us at plosone@plos.org with a request to remove this option.4. Please include captions for your Supporting Information files at the end of your manuscript, and update any in-text citations to match accordingly. Please see our Supporting Information guidelines for more information: http://journals.plos.org/plosone/s/supporting-information.

Please review your reference list to ensure that it is complete and correct. If you have cited papers that have been retracted, please include the rationale for doing so in the manuscript text, or remove these references and replace them with relevant current references. Any changes to the reference list should be mentioned in the rebuttal letter that accompanies your revised manuscript. If you need to cite a retracted article, indicate the article’s retracted status in the References list and also include a citation and full reference for the retraction notice.

Additional Editor Comments (if provided):

[Note: HTML markup is below. Please do not edit.]

Reviewers' comments:

Reviewer's Responses to Questions

**Comments to the Author**

1. If the authors have adequately addressed your comments raised in a previous round of review and you feel that this manuscript is now acceptable for publication, you may indicate that here to bypass the “Comments to the Author” section, enter your conflict of interest statement in the “Confidential to Editor” section, and submit your "Accept" recommendation.

Reviewer #3: All comments have been addressed

Reviewer #4: (No Response)

**********

2. Is the manuscript technically sound, and do the data support the conclusions?

The manuscript must describe a technically sound piece of scientific research with data that supports the conclusions. Experiments must have been conducted rigorously, with appropriate controls, replication, and sample sizes. The conclusions must be drawn appropriately based on the data presented.

Reviewer #3: Partly

Reviewer #4: Partly

**********

3. Has the statistical analysis been performed appropriately and rigorously?

Reviewer #3: Yes

Reviewer #4: N/A

**********

4. Have the authors made all data underlying the findings in their manuscript fully available?

The PLOS Data policy requires authors to make all data underlying the findings described in their manuscript fully available without restriction, with rare exception (please refer to the Data Availability Statement in the manuscript PDF file). The data should be provided as part of the manuscript or its supporting information, or deposited to a public repository. For example, in addition to summary statistics, the data points behind means, medians and variance measures should be available. If there are restrictions on publicly sharing data—e.g. participant privacy or use of data from a third party—those must be specified.

Reviewer #3: Yes

Reviewer #4: Yes

**********

5. Is the manuscript presented in an intelligible fashion and written in standard English?

PLOS ONE does not copyedit accepted manuscripts, so the language in submitted articles must be clear, correct, and unambiguous. Any typographical or grammatical errors should be corrected at revision, so please note any specific errors here.

Reviewer #3: Yes

Reviewer #4: (No Response)

**********

6. Review Comments to the Author

Please use the space provided to explain your answers to the questions above. You may also include additional comments for the author, including concerns about dual publication, research ethics, or publication ethics. (Please upload your review as an attachment if it exceeds 20,000 characters)

Reviewer #3: Congratulations to the author for choosing a pertinent topic. However the author is suggested to follow the PRISMA guidelines. The conclusion is missing which again should be written separately aligned to objectives of the review.

Likewise registration of protocol is not done, however author must indicate where the review protocol can be accessed, or state that a protocol was prepared or not also explain any amendments to information provided in the protocol.

The author is suggested to cite studies that might appear to meet the inclusion criteria, but which were excluded, and explain why they were excluded in result section.

All the best for revised submission and publication.

Reviewer #4: Review Comments: -

The authors have conducted a thorough review with clear identification of gaps in the literature and directions for future research. However, several areas could be improved to enhance the clarity and impact of the manuscript:

1.Clarification of Psychological Theories and Models: The authors mention psychological consultation and training, but it is unclear which psychological theories informed the training. It would be beneficial to emphasize the importance of consistently identifying these theoretical foundations across studies. Discussing how different theories impact outcomes could guide future research and service development.

2.Generalisability and Standardization: The discussion about generalisability could be expanded. The authors should suggest how future studies could improve on this limitation, possibly by proposing a standardized framework for the OPD Pathway. This would ensure more consistent delivery across regions and allow for better comparison and evaluation of outcomes.

3.Addressing Methodological Gaps: The manuscript acknowledges that some studies failed to meet quality standards. The authors should propose specific methodological improvements, such as encouraging the use of validated measures, increasing rigor in evaluation designs, or integrating longitudinal methods. Providing specific examples or frameworks for improving study design would enhance future research.

4.Focus on Offender Wellbeing: Offender well-being was scarcely explored in the studies reviewed. The authors could propose more detailed methodologies for incorporating well-being measures into future studies, particularly through qualitative approaches. This would make the review more actionable for researchers expanding this area.

5.Practical Recommendations for Policymakers: The "Implications for Practice and Policy" section could be strengthened by offering specific suggestions for policymakers. This could include recommending key areas where psychological training should be mandated and tools such as the Relational Security Explorer for evaluating relational security.

6.Collaborative Validation: The authors could recommend that future reviews use a more collaborative validation process involving multiple independent reviewers. This would reduce potential bias and increase the reliability of findings.

7.Expanding on Gendered Approach: The review suggests exploring gendered differences in psychological complexities. The authors could offer more detailed examples of how a gendered approach could be applied, such as examining how male and female service users respond differently to the OPD Pathway and proposing specific outcome measures that may vary by gender.

8.Risk and Reconviction Data: The manuscript highlights limited data on risk and reconviction outcomes, which are critical indicators of program success. The authors could explore why this gap exists and suggest ways to encourage future research to focus on these outcomes. A more detailed analysis or explanation for the limited data would improve the discussion.

9.Workforce Outcomes: The review finds improvements in workforce outcomes, such as confidence and competence, but could benefit from a more granular analysis. The authors could explore which specific components of psychological support and training were most effective, offering insights into what works best.

10.Clarify the Impact of Low-Quality Studies: Including lower-quality studies is acknowledged but should be clarified. A sensitivity analysis comparing higher- and lower-quality studies could provide more nuanced conclusions about the robustness of the evidence.

11.Enhancing the Conclusion with Practical Implications: The conclusion could be strengthened by discussing concrete implications for practitioners and policymakers. The authors could highlight what probation services should implement based on these findings and how future interventions should be designed to maximize their impact.

12.Improving the Synthesis of Quantitative and Qualitative Data: Since the review includes both quantitative and qualitative studies, the authors should provide a more integrated synthesis of the findings from these two approaches. Highlighting common themes or contrasting insights would give readers a fuller understanding of how different types of evidence contribute to the overall conclusions.

**********

7. PLOS authors have the option to publish the peer review history of their article (what does this mean? ). If published, this will include your full peer review and any attached files.

If you choose “no”, your identity will remain anonymous but your review may still be made public.

**Do you want your identity to be public for this peer review?** For information about this choice, including consent withdrawal, please see our Privacy Policy .

Reviewer #3: **Yes: ** Dr Jarina begum, professor in department of community medicine, Manipal Tata Medical College, Jamshedpur, Jharkhand

Reviewer #4: **Yes: ** Dr. Khalid Bashir

**********

[NOTE: If reviewer comments were submitted as an attachment file, they will be attached to this email and accessible via the submission site. Please log into your account, locate the manuscript record, and check for the action link "View Attachments". If this link does not appear, there are no attachment files.]

While revising your submission, please upload your figure files to the Preflight Analysis and Conversion Engine (PACE) digital diagnostic tool, https://pacev2.apexcovantage.com/ . PACE helps ensure that figures meet PLOS requirements. To use PACE, you must first register as a user. Registration is free. Then, login and navigate to the UPLOAD tab, where you will find detailed instructions on how to use the tool. If you encounter any issues or have any questions when using PACE, please email PLOS at figures@plos.org . Please note that Supporting Information files do not need this step.

Attachment Submitted filename: PLOS REVIEW DOCUMENT.docx

# Author comment: Author response to Decision Letter 2

**DOI:** 10.1371/journal.pone.0318428.r005

25 Nov 2024

Sincere thanks to both reviewers for their helpful and constructive feedback. We have endeavoured to address all comments by taking the following specific actions:

Reviewer 3

As suggested, a separate conclusion aligned to objectives of the review has been added.

While a protocol was not prepared, the lines of enquiry for this systematic review were informed directly by the OPD strategy which was published in 2015. This document has been referenced in the manuscript and a paragraph in the Methods section informs readers of where to access this strategy. The concerns around absence of a protocol are also addressed in the ‘Discussion’ section.

We have added a line to the ‘Study selection’ section to indicate the number of studies appearing to meet the criteria but excluded from the review.

Reviewer 4

1. Clarification of Psychological Theories and Models:

Specific reference to psychological models informing OPD service delivery have been made throughout the manuscript. We have also addressed the integration of models in service delivery and noted the difficulty in determining direct impact of individual models on outcomes as a result of this integration. We have, however, provided some suggestions in the ‘Future research’ section for clearer identification of theoretical approaches utilised.

2. Generalisability and Standardization:

We have expanded the discussion on generalisability and made direct suggestions for improvements to service delivery and data collection practices that will help make findings from future studies more generalisable. The reviewer’s suggestion to propose a standardised framework for the OPD Pathway was followed.

3. Addressing Methodological Gaps:

All suggestions on discussing methodological gaps were addressed and incorporated throughout the ‘Findings’ and ‘Discussion’ sections.

4. Focus on Offender Wellbeing:

More attention has been given to the discussion of wellbeing outcomes and specific suggestions for accessing more relevant wellbeing data were made.

5. Practical Recommendations for Policymakers:

The reviewer’s specific suggestions for mandating training and tools use were followed and more direct implications for policy and practice were added.

6. Collaborative Validation:

We have added the suggestion to involve independent reviewers in future reviews of this nature to the ‘Limitations in the review process’ section and have noted this would improve reliability of findings.

7. Expanding on Gendered Approach:

We have expanded on the discussion of taking a gendered approach in future studies and incorporated the reviewer’s specific suggestion to propose examining how male and female service users respond differently to the OPD Pathway.

8. Risk and Reconviction Data:

We have provided some further insights into the absence of good quality risk and reconviction data and discussed the complexities around access to this kind of information, both on a service level and a national level. Specific points around difficulties in data linking and access requirements were included to help elaborate on this issue.

9. Workforce Outcomes:

Granular analysis of workforce outcomes was not possible in this review as it was not generally clear in the published studies what aspects of training or consultation were effective. We have given some space to discussion around the interwoven nature of service delivery and the benefits of having a clearer framework against which to monitor outcomes. Linked with points 1 and 2, we have made suggestions for standardisation of services and training and better targeted outcomes monitoring. It is proposed that implementing more structured frameworks might help future studies address what works best in these services.

10. Clarify the Impact of Low-Quality Studies:

A section on sensitivity has been included in the ‘Certainty of evidence (Limitations)’ section and a brief summary table comparing the direction of effect found in high and low quality studies has been added.

11. Enhancing the Conclusion with Practical Implications:

Specific discussion points around practical implications were added and these provided suggestions for consistent service delivery which should help identify what works in this field of work. Proposals for service improvements in line with these suggestions have been made.

12. Improving the Synthesis of Quantitative and Qualitative Data:

We have noted in the ‘Limitations in the review process’ section that higher quality study design may have allowed for a better synthesis of findings across qualitative and quantitative papers but that a sophisticated synthesis of themes was not possible with the studies reviewed.

Attachment Submitted filename: Response to reviewers 2024.docx

# Peer review: Decision Letter 2

**DOI:** 10.1371/journal.pone.0318428.r006

**Authors:** Pracheth Raghuveer

16 Jan 2025

Does psychological support, training and guidance for probation practitioners lead to improved outcomes for service users and staff? A systematic review of Offender Personality Disorder (OPD) Pathway community delivery

PONE-D-23-24839R2

Dear Dr. O ' Meara,

We’re pleased to inform you that your manuscript has been judged scientifically suitable for publication and will be formally accepted for publication once it meets all outstanding technical requirements.

Within one week, you’ll receive an e-mail detailing the required amendments. When these have been addressed, you’ll receive a formal acceptance letter and your manuscript will be scheduled for publication.

An invoice will be generated when your article is formally accepted. Please note, if your institution has a publishing partnership with PLOS and your article meets the relevant criteria, all or part of your publication costs will be covered. Please make sure your user information is up-to-date by logging into Editorial Manager at Editorial Manager®  and clicking the ‘Update My Information' link at the top of the page. If you have any questions relating to publication charges, please contact our Author Billing department directly at authorbilling@plos.org.

If your institution or institutions have a press office, please notify them about your upcoming paper to help maximize its impact. If they’ll be preparing press materials, please inform our press team as soon as possible -- no later than 48 hours after receiving the formal acceptance. Your manuscript will remain under strict press embargo until 2 pm Eastern Time on the date of publication. For more information, please contact onepress@plos.org.

Kind regards,

Pracheth Raghuveer, MD, DNB

Academic Editor

PLOS ONE

Additional Editor Comments (optional):

Reviewers' comments:

Reviewer's Responses to Questions

**Comments to the Author**

1. If the authors have adequately addressed your comments raised in a previous round of review and you feel that this manuscript is now acceptable for publication, you may indicate that here to bypass the “Comments to the Author” section, enter your conflict of interest statement in the “Confidential to Editor” section, and submit your "Accept" recommendation.

Reviewer #3: All comments have been addressed

Reviewer #4: All comments have been addressed

**********

2. Is the manuscript technically sound, and do the data support the conclusions?

The manuscript must describe a technically sound piece of scientific research with data that supports the conclusions. Experiments must have been conducted rigorously, with appropriate controls, replication, and sample sizes. The conclusions must be drawn appropriately based on the data presented.

Reviewer #3: Partly

Reviewer #4: Yes

**********

3. Has the statistical analysis been performed appropriately and rigorously?

Reviewer #3: Yes

Reviewer #4: Yes

**********

4. Have the authors made all data underlying the findings in their manuscript fully available?

The PLOS Data policy requires authors to make all data underlying the findings described in their manuscript fully available without restriction, with rare exception (please refer to the Data Availability Statement in the manuscript PDF file). The data should be provided as part of the manuscript or its supporting information, or deposited to a public repository. For example, in addition to summary statistics, the data points behind means, medians and variance measures should be available. If there are restrictions on publicly sharing data—e.g. participant privacy or use of data from a third party—those must be specified.

Reviewer #3: Yes

Reviewer #4: Yes

**********

5. Is the manuscript presented in an intelligible fashion and written in standard English?

PLOS ONE does not copyedit accepted manuscripts, so the language in submitted articles must be clear, correct, and unambiguous. Any typographical or grammatical errors should be corrected at revision, so please note any specific errors here.

Reviewer #3: Yes

Reviewer #4: Yes

**********

6. Review Comments to the Author

Please use the space provided to explain your answers to the questions above. You may also include additional comments for the author, including concerns about dual publication, research ethics, or publication ethics. (Please upload your review as an attachment if it exceeds 20,000 characters)

Reviewer #3: Dear Author,

The revised draft of the manuscript looks good to go. However, few more suggestions to improve it further are mentioned below: 1. Organize the content in more comprehensive manner 2. Try to build the article with no repetition of information, in appropriate sequence of events in the study.3. Although justified, it was recommended to do Prospero registrations for all SRMA in future. All the best for final publication.

Reviewer #4: I have thoroughly reviewed the revised manuscript and am pleased to note that the authors have addressed all my queries and suggestions in detail. They have demonstrated a clear effort to incorporate feedback, enhancing the quality and relevance of the work.

Key improvements include:

Clarification of the integration of psychological theories and models in OPD service delivery.

Expanded discussion on generalizability and suggestions for a standardized framework.

Addressing methodological gaps and limitations comprehensively.

Greater emphasis on offender wellbeing and gendered approaches.

Practical recommendations for policymakers and improved focus on workforce outcomes.

Enhanced synthesis of qualitative and quantitative data.

The authors have also acknowledged the limitations of their review and provided thoughtful recommendations for future research. These additions significantly strengthen the manuscript’s contribution to the field.

Based on these revisions, I am confident in recommending this manuscript for acceptance. It is a valuable resource that will undoubtedly contribute to advancing understanding and practice in the targeted area of study.

**********

7. PLOS authors have the option to publish the peer review history of their article (what does this mean? ). If published, this will include your full peer review and any attached files.

If you choose “no”, your identity will remain anonymous but your review may still be made public.

**Do you want your identity to be public for this peer review?** For information about this choice, including consent withdrawal, please see our Privacy Policy .

Reviewer #3: **Yes: ** Dr Jarina begum

Reviewer #4: **Yes: ** Dr. Khalid Bashir, MBBS, MD Email ID: - drkhalidbashir.s@gmail.com

**********

Attachment Submitted filename: REVIEWER COMMENTS plos.docx

# Editor report: Acceptance letter

**DOI:** 10.1371/journal.pone.0318428.r007

**Authors:** Pracheth Raghuveer

PONE-D-23-24839R2

PLOS ONE

Dear Dr. O'Meara,

I'm pleased to inform you that your manuscript has been deemed suitable for publication in PLOS ONE. Congratulations! Your manuscript is now being handed over to our production team.

At this stage, our production department will prepare your paper for publication. This includes ensuring the following:

* All references, tables, and figures are properly cited

* All relevant supporting information is included in the manuscript submission,

* There are no issues that prevent the paper from being properly typeset

If revisions are needed, the production department will contact you directly to resolve them. If no revisions are needed, you will receive an email when the publication date has been set. At this time, we do not offer pre-publication proofs to authors during production of the accepted work. Please keep in mind that we are working through a large volume of accepted articles, so please give us a few weeks to review your paper and let you know the next and final steps.

Lastly, if your institution or institutions have a press office, please let them know about your upcoming paper now to help maximize its impact. If they'll be preparing press materials, please inform our press team within the next 48 hours. Your manuscript will remain under strict press embargo until 2 pm Eastern Time on the date of publication. For more information, please contact onepress@plos.org.

If we can help with anything else, please email us at customercare@plos.org.

Thank you for submitting your work to PLOS ONE and supporting open access.

Kind regards,

PLOS ONE Editorial Office Staff

on behalf of

Dr. Pracheth Raghuveer

Academic Editor

PLOS ONE
